# Exploring condom use decision-making among adolescents: the synergistic role of affective and rational processes

**DOI:** 10.1186/s12889-021-11926-y

**Published:** 2021-10-19

**Authors:** Eugene L. Davids, Yanga Zembe, Petrus J. de Vries, Catherine Mathews, Alison Swartz

**Affiliations:** 1 Adolescent Health Research Unit, Division of Child & Adolescent Psychiatry, University of Cape Town, 46 Sawkins Road, Rondebosch, 7700 South Africa; 2 Independent Institute of Education, Varsity College, Cape Town, South Africa; 3 School of Built Environment & Development Studies, University of Kwazulu-Natal, Durban, South Africa; 4 Health Systems Research Unit, South African Medical Research Council, Cape Town, South Africa; 5 Division of Social & Behavioural Sciences, School of Public Health & Family Medicine, University of Cape Town, Cape Town, South Africa

## Abstract

**Background:**

Condom use remains the most effective behavioural method for the prevention of HIV and unplanned pregnancies. However, condom use remains inconsistent among young people. Exploring the condom use decision-making processes that adolescents engage in might provide information that would assist in the prevention of many challenges related to poor sexual and reproductive health outcomes. This study therefore aimed to explore the factors that influenced decision-making about sexual debut and condom use of adolescents from two schools in the Western Cape, South Africa.

**Methods:**

A sample of 16 adolescents were selected using purposive sampling. Data were collected using semi-structured, individual interviews. Thematic analysis was used to analyse the data generated.

**Results:**

The link between sexual debut and affective processes was frequently discussed in condom use decision-making. Decisions about sexual debut were influenced by the belief that sex was a perceived symbol of ‘true love’ on the one hand, and respect for perceived parental expectations of age-appropriate sex, on the other. Condom use decision-making was shaped by adolescents’ concerns about their future and lack of stability in their lives. Adolescents’ fears of pregnancy, parenthood and disease shaped their condom use decision-making. It became evident that rational and affective decision-making in condom use choice were not mutually exclusive, but that these processes happened simultaneously.

**Conclusions:**

The study highlighted the role of affective states as part of the process of examining alternatives when deciding to use a condom or not. Interventions to strengthen condom use decision-making should therefore incorporate not only rational but also affective processes to improve adolescent sexual and reproductive outcomes.

## Background

Globally, 1.6 million adolescents are currently living with HIV [1] of which two-thirds live in sub-Saharan Africa (SSA) [2]. Adolescent girls between ages 15 and 19 account for most of the new HIV infections in SSA [3]. Condom use remains among the most effective behavioural methods for the prevention of HIV and unplanned pregnancies [4]. The prevalence of condom non-use among adolescents in SSA is estimated to be 60% [5]. In South Africa, even though many young people are sexually active [6–8], condom use is inconsistent, with a reported rate of condom use inconsistency at 46–55% [9, 10]. Recent findings presented in the South African Demographic and Health Survey, suggests that 50% of young women and 66% of young men reported having sex before the age of 18, of which 4.6% of young women and 20.7% of young men had more than 2 sexual partners in the 12 months before the survey. Only 62.3% of these young women and 72.9% of these young men reported using a condom [11].

Adolescents are at risk of poor sexual and reproductive health outcomes often due to a combination of early, unprotected, or coerced sex, and the lack of adolescent-friendly sexual and reproductive health services [12–14]. These outcomes include unintended pregnancies, abortion, as well as sexually transmitted infections (STIs) including HIV [2]. HIV acquisition and transmission among adolescents most often results from unprotected sex or inconsistent condom use. While it is difficult to pinpoint exactly what shapes the choice to engage in unprotected sex or inconsistent condom use, we know that condom use decision-making happens in an environment that is changing rapidly [2, 15]. These changes include the increase in access to and use of technology and social media which is often associated with exposure to sexually-explicit social media accounts and sexually-orientated reality television predicting willingness to engage in casual sex [16]. Diverse perspectives on childbearing and marriage including shifts in cultural, sociological and demographic views of childbearing and marriage have seen an increase in births outside of marriage [17], as well as the onset of puberty and changing social roles such as changes in the age of onset which influences adolescent risk behaviour and the increase in premarital sex among young men and women [11, 18].

**Fig. 1:**
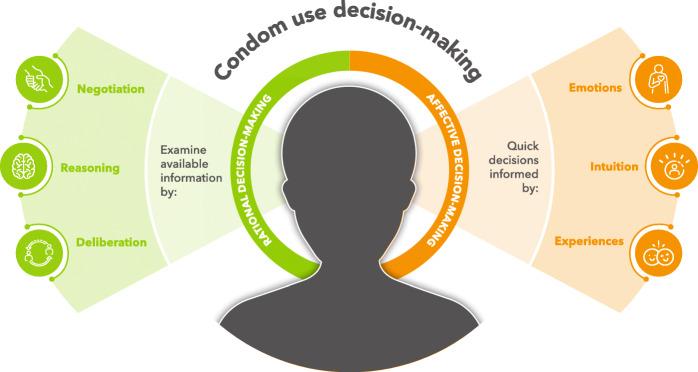
Adolescent decision-making is often informed by two theoretical perspectives, namely affective and rational decision-making

Adolescent decision-making processes have primarily been examined from two theoretical perspectives – (i) the affective decision-making (referred to as emotion-based decision-making) and (ii) a rational decision-making (referred to as fact / information-based decision-making) perspective as outlined in Fig. 1 [19]. The affective perspective on decision-making is concerned with the role of emotions and intuition in the decision-making process, while the rational perspective examines the role of negotiation and reasoning in coming to a decision [19, 20]. Ferrer and Mendes [21] have highlighted that the role of affective states in making and shaping decisions, particularly related to health and behaviour, remains poorly understood. The process of making decisions, whether informed by affective states, available information, or both, may of course differ from individual to individual – which is often referred to as the decision-making style [22].

An adolescent’s decision-making style could be protective against adverse health-related consequences. Decision-making styles could also increase the risk of adverse health-related consequences. It is important to acknowledge that there may be situations in which an adolescent’s agency is constrained in such a way that does not allow them to make choices. For example, choices related to condom use would be constrained in situations where sex is coerced or intimate partner/sexual violence is occurring. Adolescent choices might be shaped by sociocultural factors which would impact their decisions related to sexual and reproductive health – such as gender, power, cultural and religious views. Empowering adolescents to make good decisions to promote sexual and reproductive health is important, but the social and structural factors that constrain their agency must also be considered when exploring how best to encourage protective behaviours in this sphere. A more careful understanding of decision-making would assist in identifying the processes or mechanisms to improve decision-making.

Exploring condom use decision-making processes might provide information that would assist in preventing many challenges related to poor sexual and reproductive health outcomes of adolescents. The process of knowing when to ‘start, stop or change’ condoms and other contraceptive methods is complex for adolescents [23]. The complexity which emerges in these decisions is as a result of the interpersonal, community and macro-social influences [24].

Interventions leading to favourable behavioural outcomes that promote sexual and reproductive health are needed, particularly as young people make up a large proportion of the global population [2]. Many sexual and reproductive health interventions have focused on increasing knowledge about sexual and reproductive health risks, which is still important as there is an urgent need to inform young people, but it will not be sufficient. Importantly, few sexual and reproductive health studies have qualitatively examined what drives adolescents’ decision-making process before a behavioural outcome is selected. Examining the decision-making process and its influences on sexual behaviour could inform health-related interventions. Given these knowledge gaps, the current study aimed to explore the factors that influence adolescents’ sexual debut and condom use decision-making in the Western Cape, South Africa, with a particular focus on the affective and rational factors that shape this decision-making. The current study is a sub-study of a larger research project that aims to better understand adolescent health decision-making.

## Methods

### Design

The study employed a qualitative descriptive approach to examine the factors that influence adolescent condom use decision-making. The consolidated criteria for reporting qualitative research (COREQ) guidance for reporting qualitative research has been applied in presenting the study.

### Recruitment & participants

Participants for the study were recruited from two public schools in the Western Cape province of South Africa. An initial list of all public schools was generated and stratified on the basis of socio-economic status, using school fees as a proxy. Three schools were randomly selected in each of the strata (no school fees, ZAR 1 – ZAR 1500 per annum and above ZAR 1500 per annum), of which one school in each strata were invited to participate in the study. Of the nine schools invited three initially agreed, but ultimately only two schools agreed to participate. The seven schools who did not participate in the study were due to other active research commitments in the various schools.

The final sample included 16 participants from these two public schools, selected due to pragmatic considerations and previous study sample sizes examining adolescent health. Participants were purposively selected to include an equal split between sex, socio-economic status and developmental phases (summarised in Table 1 below). The developmental phase was categorised into early and late adolescence, using both age and educational level/grade as an indicator. Participants who were in the eighth grade in secondary school were categorised as being in the developmental phase of early adolescence, and those in the eleventh grade as late adolescence. The rationale behind this selection of participants was to see if there were differences in responses about condom use decision-making on the basis of these demographic descriptors.

**Table 1:** Study sample demographic details (*n* = 16)

Demographic descriptor(s)	Frequency
**Age (Mean)**	15.38 years old
Age range	13–18 years old
**Sex**	
Male	8
Female	8
**Developmental phase**	
Early adolescence (8th Grade)	8
Late adolescence (11th Grade)	8
**Socio-economic status**	
Low socio-economic setting	8
High socio-economic setting	8

### Procedures

The study was a sub-study of a larger research project, both protocols received ethical clearance from the University of Cape Town Faculty of Health Sciences Human Research Ethics Committee (HREC References: 301/2017 & 356/2019) as well as permission from the Western Cape Education Department (WCED) to access schools within the Western Cape (WCED Reference: 20170706–2719). After appropriate approvals were granted, the first author (ELD) made contact with principals at the selected schools and set up initial meetings with the principal and relevant teaching staff at the selected schools to invite them to partake in the study. Students were then invited to participate in the study and given an information sheet, parental consent, and student assent forms. Following receipt of the completed consent and assent forms, dates and times were selected by the school to ensure that data collection would be least disruptive to the normal school day. Only researcher contact details were shared with participants, and no prior relationships were established before the study commenced.

### Data generation

Semi-structured interviews were conducted by two researchers, on the school premises. The first author conducted all interviews with English-speaking participants (*n* = 10), and an independent second researcher (SM) conducted all interviews with isiXhosa-speaking participants (*n* = 6). The first author was male, with an educational background in psychology and public health and his highest qualification was a Ph.D. He was a post-doctoral research fellow at the time of the study with experience in adolescent health research. The independent researcher was a female, with an educational background in psychiatric nursing and holds a Ph.D. She was employed as a senior psychiatric nurse at the time of the study. All interviews were audio-recorded, and the length of the interviews ranged between 45 min to 1 hour. Various vignettes about adolescents facing various health-related decisions were used to guide the interviews but this article focussed on decisions related to sexual debut and condom use. Using a vignette afforded participants the opportunity to consider their hypothetical responses even if they had never engaged in sexual intercourse, condom use negotiation and/or other health-related decisions (see Table 2 for vignette and examples of specific questions related to condom use decision-making). During interviews, both researchers conducting the interviews kept brief notes and also met to debrief following each interview to discuss the emerging thoughts, interpretations and when no new codes, themes and insights emerged from the analysis [25]. No additional or repeat interviews were conducted with participants.

**Table 2:** Vignette and specific questions used to elicit information about condom use decision-making

*Vignette*	
Craig and Ntombi have been friends for a long time. They have decided to take their relationship to the next level. One night at a party Ntombi is offered vodka and beers to drink. She sees all the other friends at the party drinking vodka, and decides to drink the vodka instead of the beer. After having several rounds of vodka she decides to go outside at the party and get some fresh air. When Ntombi gets outside she is offered a smoke – she isn’t exactly sure what it is but takes it and takes a few puffs. Later she realises that it is dagga that she was smoking. As the party goes on – she starts feeling a bit funny. She tells Craig and he decides to take her to his place. His parents are away for the weekend. Later Craig and Ntombi decide to engage in sex. Craig does not want to use a condom but Ntombi insists that he should use one. While the two decide on what to do, they start arguing Craig starts beating Ntombi.	
*Examples of questions*	
‘How about Ntombi (a character in the vignette) wanting to use a condom and Craig (another character in the vignette) not? What would you or your friends do in a situation like this?’, ‘What would make you decide to choose that option?’, ‘How would you go about selecting that option?’, ‘What if you chose the other option; what would have played a role in you selecting that?’, and ‘Tell me about the process that you would go through if you were to choose that option?’	

### Data analysis

English interviews were transcribed verbatim by an independent transcriber and checked by the first author. Interviews conducted in isiXhosa were translated and transcribed into English by an independent bilingual transcriber, and checked by the isiXhosa-speaking interviewer for accuracy.

The transcribed interviews were analysed by the first author, using a combination of manual coding and NVivo 11 using the steps as outlined by Braun and Clarke [26] to conduct thematic analysis. The transcribed interviews were coded using inductive coding, allowing codes and themes to emerge from the data guided by the process of thematic analysis as outlined by Braun and Clarke [26]. Twenty-eight per cent (*n* = 5) of the transcribed interviews were coded and checked by a senior qualitative researcher (YZ). The codes and themes emerged through the analyses by the first author and the senior qualitative researcher were then compared and discussed. Themes and the substantiated quotations from the transcribed interviews were also discussed and refined among peers in a structured, peer-reviewed plenary and with two other senior authors (AS, CM). Participants did not provide feedback on themes generated.

### Scientific rigour

The integrity and scientific rigour of the study was promoted throughout using the following strategies: (i) To ensure credibility in the study, investigator triangulation was performed where the data and interpretations were generated by more than one researcher throughout the coding process [27, 28]. (ii) Trustworthiness, dependability and confirmability of the research study was made possible through rich descriptions and transparency in the steps that were taken in the study from the development of the larger study to the presentation of the findings [27]. This was further promoted through the justification of how the method employed was best suited for the intended study aim [27, 28]. (iii) Researchers often met to discuss and reflect on their interviews using their field notes and reflections which encouraged reflexivity in the study [27].

## Results

**Fig. 2:**
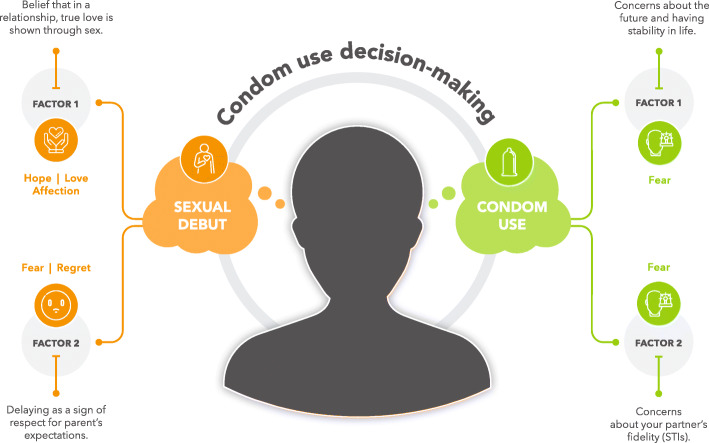
Adolescent condom use decision-making is often informed by discussions related to sexual debut and condom use. Decisions related to sexual debut is informed by the belief that when in a relationship true love is shown through having sex which is informed by the affective component of hope, love and affection. Sexual debut decisions are also informed by the perceptions of parents related to age-appropriate sex which is shaped by the affective component of fear and regret. Decisions related to condom use is informed by concerns about the future (such as becoming pregnant or becoming a parent) which is informed by the affective component of fear. Condom use decisions are also informed by concerns about partner fidelity related to contracting STIs which is shaped by the affective component of fear too

When exploring the factors that influence adolescents’ condom use decision-making, sexual debut emerged as the most prevalent scenario informing condom use decisions. Results were therefore grouped according to themes that emerged for (i) sexual debut and (ii) condom use decision-making, respectively (see Fig. 2). The sample included 16 adolescents with a mean age of 15 years, ranging from 13 to 18 years. There was an equal split in terms of sex, socio-economic status and developmental phase (See Table 1). The themes which emerged showed no differences for condom use decision-making among adolescents in terms of the demographic descriptors of sex, socio-economic status and developmental phases.

### Decision-making about sexual debut

When speaking about condom use decision-making, adolescents often made reference to sexual debut and how they navigated their thought processes about it. Adolescent sexual debut was repeatedly shown to be influenced by adolescents’ belief that relationships were about showing true love for one’s romantic partner through sex on the one hand, and on the other hand, that sexual debut should be delayed as a sign of respect for parents’ expectations and rules about the appropriate age to have sex. These two themes are further detailed below.

#### Sex as a symbol/manifestation of true love

Adolescence has often been considered as a developmental period of exploration and discovery, with sexual development highlighted as an integral part of the phase. When adolescents referred to relationships and the role of sex within them, it was clear that they considered sex to be an important mechanism by which ‘true love’ can be shown to a romantic partner. The link between sex and true love framed their decision-making regarding sexual debut:

‘I think a lot of people would say it’s in the way to show like their true love for each other … ’ (Female, Age 17, High SES, Late Adolescence, Interview ID: MPH7).

Further, it was not just any kind of sex that was framed as proof of true love; the adolescents specifically talked about demonstrating true love through sex that involved the use of condoms. Condoms were spoken about as euphemisms for the sex act rather than explicitly speaking about sex:

‘They are in a relationship *mos* [colloquial word for right or OK] … If she is honest that she loves me and if I can also see that she loves me then I was going to say so … Yes, you were going to take it [referring to a condom] because the day you [are] taking your relationship to the next level … ’ (Male, Age 15, Low SES, Early Adolescence, Interview ID: LHS3).

The decision-making style or process in which adolescents engaged when exploring sexual debut was characterized by societal expectations about what a romantic relationship entails. The adolescents appeared to believe that being in a relationship brought with it the expectation that they show and prove their love and affection for their partner. In this scenario, often the idea of love and affection was equated with having sex, which was considered as part of the set of expectations that characterize romantic relationships. One participant indicated this expectation by stating that adolescents should engage in sex because ‘they are in a relationship’ (Male, Age 14, Low SES, Early Adolescence, Interview ID: LHS7). Some of the adolescents viewed the decision-making process to be about the hope and the expectation of showing true love within their relationship. Showing true love was viewed as a gateway to sex within the relationship.

#### Delaying sexual debut as a sign of respect for parents’ expectations and rules

Some participants did not view sexual debut as being an option for them because of their immature age. As two participants put it:

‘It is dangerous to have sex … ’ (Female, Age 14, Low SES, Early Adolescence, Interview ID: LHS4)

‘ … because it’s just … it’s not on the table at the moment’ (Female, Age 17, High SES, Late Adolescence, Interview ID: MPH8).

Some of the adolescents explained that delaying sexual debut had to do with their observation of their parents’ teachings that sex was not to be entertained at this stage of development:

‘ … because the way I’m raised, my parents taught me that it is for after marriage … ’ (Female, Age 17, High SES, Late Adolescence, Interview ID: MPH7)

Thus, consideration of their parents’ rules and the perceptions of what is right and wrong framed some of the adolescents’ decision-making regarding when to have sex. Further, subtle indications of the emotion of fear of consequences coupled with regret for having ‘wronged’ parents were evident in the adolescents’ considerations of sex and the choices around it in the context of their upbringing. Fears of what their parents would do if they found out they had engaged in sex during their adolescence, held some of the adolescents back from engaging in early sexual debut as captured by the following quote:

‘What is my parents going to do if they found out?’ (Female, Age 13, High SES, Early Adolescence, Interview ID: MPH3).

### Condom use decision-making

Adolescents’ condom use decision-making was characterized by concerns about the future and fear sexual and reproductive consequences such as becoming pregnant or being infected with a sexually transmitted disease.

#### Concerns about the future and fear of perceived negative consequences

When adolescents explored condom use decision-making, it was clear that this was shaped by anxiety and concern about their future. This is in line with evidence of how affective states or emotions such as anxiety drive the examination of the best alternatives available to address the situation at hand. When considering condom use, adolescents expressed their concerns about their future and the need to have stability. Stability to these participants took the form of access to financial resources, pursuing further education and securing a job:

‘ … I also don’t want to put myself in that kind of situation, because I still want to study more and there’s no need to rush, there’s still time. First, I finish my studying, have my own money, … first I have to sort out my life, then I can start doing whatever I want … ’ (Female, Age 14, Low SES, Early Adolescence, Interview ID: LHS1).

‘Fear … also you have to think about your future, your surroundings, and what other people think of you … That’s what most people use to make decisions’ (Male, Age 13, High SES, Early Adolescence, Interview ID: MPH2)

The adolescents decision-making process regarding condom use was clearly marked by anxieties about the future, which they thought would be compromised if they engaged in condomless sex and consequently contracted a sexually transmitted infection or became pregnant. Some adolescents put it this way:

‘ … Because I learned a lot of things in school about contraceptive[s]. How they can protect you from various STDs and prevent you from getting a baby and all those things … ’ (Male, Age 16, High SES, Late Adolescence, Interview ID: MPH5)

‘You don’t want that [referring to becoming a parent or contracting HIV] for yourself … so you will use the best thing, a condom … ’ (Female, Age 13, High SES, Early Adolescence, Interview ID: MPH3).

‘I was going to be influenced by the things I have seen happening because of unprotected sex. Before I even speak about HIV, after impregnating a person life becomes difficult … there is a possibility that you can be infected with HIV and STI’s or STD’s.’ (Male, Age 16, Low SES, Late Adolescence, Interview ID: LHS6)

The adolescents’ fears that engaging in sex without condoms would lead to sexually transmitted infections was informed by uncertainties about partner fidelity; this consideration hugely influenced their decision-making regarding condom use:

‘I was going to say [yes to the] condom because you were friends *mos* [colloquial word for right or OK] at first and you don’t know how many girls did he sleep with. And even now that you are together you don’t know how many girls he has outside, so I was going to say condom because I don’t want to get infected. Because he is the only one that knows whom he sleeps with and there are diseases out there. So, I was going to say condom always.’ (Female; Age 17; Low SES; Late Adolescence, Interview ID: LHS5).

The role of fear of undesirable sexual and reproductive consequences in influencing the condom use decision-making process was apparent in many of the remarks made by participants in the study:

‘[I] wouldn’t have agreed to have sex without a condom because that’s dangerous, maybe you can fall pregnant or be infected with HIV so I wouldn’t have agreed without a condom.’ (Female; Age 14; Low SES; Early Adolescence, Interview ID: LHS4).

Another participant said:

‘They are not like, grown up enough to raise a child, when they are still children. That is a lot of the thinking of teens … I would always also say, we should use a condom rather, because it will increase the chance of [not] falling pregnant’ (Female; Age 17; High SES; Late Adolescence, Interview ID: MPH7).

The fear of raising and providing for a child was also highlighted by another participant when he remarked that he would opt for using a condom:

‘ … [Yes], fear for not being able to provide for the child … ’ (Male, Age 13, High SES, Early Adolescence, Interview ID: MPH2)

Adolescents synonymously discussed sexual debut when referring to condom use decision-making. The link between sexual debut and condom use decision-making wasn’t fully explored in the current study but previous studies suggest that early sexual debut had lower odds of condom use as part of the decision-making process [29], while condom use at sexual debut has been associated with condom use at recent sexual intercourse among adolescents [30].

## Discussion

Given the importance of condom use as a key behavioural strategy to reduce the transmission of HIV, other STIs and unplanned pregnancies [4] the study aimed to explore the factors that influence adolescents’ sexual debut and condom use decision-making in the Western Cape, South Africa.

Health-related decision-making is often viewed from one of two perspectives, the rational or affective perspective. Most interventions aimed at adolescent sexual and reproductive health are informed by the rational perspective through the provision of information, with very few informed by the affective perspective. The role of affective states in making and shaping decisions, particularly related to sexual and reproductive health, remains poorly understood. Although the study aimed to explore the factors that influence adolescents sexual debut and condom use decision-making broadly, in the interviews and during data analysis, the rational and affective perspectives emerged as the definitive decision making influences. Better understanding of the decision-making process would assist policies and programmes to promote and empower adolescents to make better decisions to promote sexual and reproductive health.

The qualitative study explored the process of decision-making using a vignette-based methodology as a proxy for ‘real-life’ decision-making. The findings suggest the presence of *both* the affective and rational influences on decision-making and that these potentially interactedwhen reaching a decision.

The results suggest that sexual debut was often discussed when exploring conversations about condom use decision-making among the adolescents in this study. The conversations about sexual debut might have emerged among the adolescents who were sexually mature but did not want to be seen as already having sex. Sexually mature could be defined as whether the adolescent has ever had sex. On the contrary, the recurrent theme of sexual debut when discussing condom use might have been due to some adolescents in the study not being sexually mature. No data were collected about adolescent sexual behaviour which might have informed the decision-making and discussions linking sexual debut to condom use. Previous studies suggest, that later sexual debut was often associated with condom use, while earlier sexual debut was synonymous with less thought and use of condoms in the decision-making process [29]. The findings in the current study highlight the associations between sexual debut and condom use decision-making that have been found in previous studies [29, 30]. The role of fear, however, was also seen as driving the decision-making process of rationally examining the alternatives when a decision needed to be taken about using a condom or not.

Scholars have called attention to the importance of understanding and exploring adolescent sexual decision-making [31] particularly related to condom use to inform HIV and pregnancy prevention interventions. Through exploring the findings of this study, it became clear that social expectancies (as shown in Fig. 2), parental influence (fear and regret associated with parental perceptions of age-appropriate sexual behaviour) and the fear of undesirable future outcomes (anxiety and fear related to stability in life, becoming parents and contracting a STI) were integral to adolescent condom use decision-making.

Using the affective decision-making perspective, values, expectancies and emotions are key when examining adolescent risk behaviour [31]. Evaluating positive and negative outcomes of behaviour during the decision-making process, called expectancies, is important to explore as adolescents exhibit agency in their choices around health-related decision-making [31]. Findings in this study confirmed the assumption that negative expectancies, such as becoming pregnant, becoming a parent or contracting HIV or other related sexual transmitted infections (that emerged in the theme related to *concerns about the future and fear of perceived negative consequences*) [31] are often shaped by the values held by adolescents.

In addition to the values and expectancies, the findings suggest that emotions and affective states, including hope, love, anticipated regret, and fear, were important factors in adolescent decision-making (seen in the themes related to sexual debut). Affective states are often ignored in health-related decision-making and thus are not always incorporated into the theoretical frameworks that underpin many health-related interventions [21]. Affective states such as motivation, emotion, and stress, to name but a few are important when making decisions about health [21]. Adolescents are making decisions about emotionally laden issues [21]. They talk about things in relation to how they think or expect they may feel. We tend to ignore emotions in interventions/research because of social constructions of emotions and the self as being vulnerable which is disempowering and perpetuates stigma [32]. Instead, we focus on risk perception, knowledge and attitudes [21], which are important too. We could reshape our interventions/research by understanding the role of emotion and affective states in health-related decision-making and behavioural outcomes.

The themes suggest the important role of an evaluation of the alternatives but also the role of affective states in driving the outcomes of such evaluations, that are often ignored in health promotion and prevention interventions. The themes related to condom use decision-making also highlighted the role of fear in the decision-making process. Fear has often been cited as influencing the process of making sense of available information which is important to making a decision. Diminished attention is paid to the risk-related information in the presence of fear. However, others have believed that the presence of fear is associated with the awareness of risk related to the potential alternatives to be chosen in the health-related decision-making process [33].

Guilamo-Ramos and colleagues [34, 35] found that adolescents were concerned about the implications and consequences that engaging in risky sexual behaviour would have on those they interact with socially, like peers, parents and neighbours. These concerns about the implications and consequences of sexual behaviour were also seen among adolescents in the current study where there were concerns about pregnancy, parenthood and disease infection. Anticipation of regret as an affective state which pointed to concerns about the social implications and consequences of sexual behaviour, was a common underlying factor that influenced how adolescents thought about sexual debut. These findings highlight not only the importance that adolescents may place on values as part of their sexual debut and condom use decision-making processes, but also how these values inform some of the expectancies or outcomes regarding condom use or non-use.

### Implications for public health interventions

Public health interventions aimed at promoting sexual and reproductive health of adolescents should not only consider adolescents as being rational young people who evaluate the available alternatives based on the knowledge shared in many sexual and reproductive health interventions. It is evident that when faced with a situation in which a choice needs to be taken around condom use or non-use, decisions are also guided by various emotions and affective states. The result suggests that these two forms of decision-making (rational and affective) are not mutually exclusive and could be happening simultaneously during the condom use decision-making process. Many sexual and reproductive health interventions that have focused on cognitive and behavioural models have seen an increase in knowledge related to sexual risk [34], but have not seen a change in behavioural outcomes. Perhaps by taking into account affect and how it might work in tandem with knowledge when thinking about adolescent sexual decision-making could be the shift in behavioural outcomes. There is a need for interventions that are based on both rational and affective decision-making processes which are central to the decision-making styles used by adolescents when making decisions around condom use and risky sexual behaviour [21, 31, 35, 36]. Interventions which consider both rational and affective decision-making processes would be categorised as behavioural change interventions stemming from motivation [37].

Behaviour change interventions aimed at improving condom use decision-making should, therefore, be multi-faceted with a combination of education (information to facilitate behaviour change), persuasion (apply communication strategies to prompt emotions or spark action), incentivisation (provide an anticipated reward), coercion (bringing about anticipated punishment or cost for actions against targeted behaviour change), environmental restructuring (foster change within the social or physical environment), modelling (have individuals to emulate or aspire to be) and enablement (provide support to increase the targeted behaviour change) components as adapted from Michie, van Stralen and West [37] Behaviour Change Wheel. Emotions and affective states have been shown to play an important role in the condom use decision-making process, addressing the gap, as highlighted by Ferrer and Mendes [21], for better understanding of emotions and affective states in health decision-making to inform intervention development and implementation [21].

In addition, interventions should consider the social ecology of adolescents, as the influence of parents and social expectations emerged as important factors in the decision making process regarding sexual debut in our study. DiClemente, Salazar and Crosby [38] have supported the notion of interventions that are ecological – considering the various levels of causation or influence in sexual debut and condom use choices – suggesting that these are effective and promote the adoption of less risky outcomes. Therefore, complex interventions that consider adolescents, relationships with others, peers, family and the community are needed which inform decision-making processes around condom use decision-making. These complex interventions would also address the different levels of causation or influence in the decision-making process which is furthermore shaped by society, culture, values, economic and other social factors [39]. Interventions might also consider role-playing situations or behavioural rehearsal in which sex and condom use negotiations take place which might inform adolescent decision-making but also decision-making competence and self-efficacy. Decision-making competence and self-efficacy have been associated with better decisional outcomes [40–42].

### Implications for further research

Understanding how adolescents make decisions about health-related behavioural outcomes should be further examined to identify and understand whether values, expectancies, anticipated future outcomes and emotions are central to the decision-making process engaged. Being able to understand and identify whether these rational and affective appraisals are part of the decision-making processes across all health-related decisions, could inform future public health interventions which are aimed at primary and secondary prevention.

### Limitations

One of the limitations of the study related specifically to the limitations inherent in the vignettes used to elicit information from the participants about their condom use decision-making: (i) the characters in the vignettes had heterosexual relationships and thus did not portray condom use among adolescents in same-sex relationships, (ii) the vignettes also failed to consider the use of other contraceptives which might have played a role in the perceptions towards condom use and thus impacted on the adolescent’s decision-making around condom use, (iii) the estimated control associated with understanding the hypothetical versus the real experience of adolescent agency in making condom use decisions might be difficult to ascertain through the use of vignettes. In addition, no data were collected about participant sexual behaviour, making the comparison between condom use and sexual debut decision-making difficult between those who have engaged in sex or not.

## Conclusion

When examining the factors that shaped condom use decision-making among adolescents it became evident that sexual debut is central to the conversations about condom use. This study highlighted the importance of the role of affective states as part of the process of examining the alternatives when making a decision about condom use or non-use. Interventions are needed that examine both the role of affective (emotion-based) and rational (knowledge-based) processes as part of the decision-making process to inform adolescent sexual and reproductive health outcomes. The factors that shape adolescent condom use decision-making as highlighted in the study could inform sexual and reproductive health interventions to promote behaviour change and reduce HIV incidence and unintended pregnancies among adolescents.

## Data Availability

The data generated during the study are not publicly available but are available from the corresponding author on reasonable request.
